# Targeting liver metastases in uveal melanoma: ATX-LPA mediated immunosuppression and novel therapeutic approaches

**DOI:** 10.3389/fimmu.2026.1829486

**Published:** 2026-06-18

**Authors:** Jacqueline A. Turner, Marc D’Antonio, Heather N. Montane, Morgan MacBeth, Elizabeth Katsnelson, William A. Robinson, Richard P. Tobin, Kasey L. Couts, Svetomir N. Markovic, Raul M. Torres

**Affiliations:** 1 Department of Internal Medicine, Mayo Clinic College of Medicine and Science, Rochester, MN, United States; 2 Department of Immunology and Microbiology, University of Colorado School of Medicine, Aurora, CO, United States; 3 Department of Oncology, Mayo Clinic College of Medicine and Science, Rochester, MN, United States; 4 Division of Medical Oncology, University of Colorado School of Medicine, Aurora, CO, United States; 5 Division of Surgical Oncology, Department of Surgery, University of Colorado School of Medicine, Aurora, CO, United States

**Keywords:** autotaxin, immunity, liver, lysophosphatidic acid, metastases

## Abstract

**Introduction:**

Uveal melanoma has a marked tropism for the liver where immune tolerance facilitates metastatic progression and resistance to immunotherapy. Impaired CD8^+^ T cell immunosurveillance is a central determinant of disease progression in uveal melanoma, yet the underlying mechanisms driving liver-mediated immune subpression and hepatic metastasis are not fully understood. Here, we investigate molecular pathways that regulated hepatic CD8^+^ T cell function and evaluate clinical outcomes associated with liver-directed therapy.

**Methods:**

Expression of autotaxin (ATX), the expression responsible for generating lysophosphatidic acid (LPA), was evaluated in hepatic resident cell populations. ATX transcript levels were assessed in CD8^+^ T cells isolated from hepatic metastases and compared with matched peripheral blood and healthy donor control. Antigen-specific effector CD8^+^ T cells were used to characterize downstream signaling responses to LPA stimulation. Functional studies evaluating cytokine production during chronic antigen exposure were performed using CD8^+^ T cells from LPA receptor 5-deficient (*Lpar5^-/-^*) mice.

**Results:**

We identify the autotaxin-lysophosphatidic acid (ATX-LPA) axis as a prominent immunoregulatory pathway in the metastatic hepatic tumor microenvironment. ATX, the enzyme responsible for LPA production, is constitutively expressed by hepatic resident cells, is detectable on Kupffer cells and is associated with increased immunosubpressive markers. CD8^+^ T cells isolated from hepatic metastasis in a patient with uveal melanoma display markedly elevated ATX transcripts compared to matched peripheral blood (>5 log_2_ fold change) and healthy controls. Using antigen-specific effector CD8^+^ T cells, we demonstrate LPA signaling induces low level, persistent ERK phosphorylation distinct from canonical antigen-induced TCR signaling. Genetic deletion of the LPA receptor 5 (*Lpar5^-/-^*) on CD8^+^ T cells restores CD8 T cell percentages and cytokine function during persistent antigen stimulation in the presence of LPA.

**Discussion:**

These findings define an ATX-LPA mechanisms of dysfunctional ERK signaling that may contribute to hepatic immune subpression in uveal melanoma. We highlight this pathway as a rational therapeutic target alongside liver-directed clinical interventions.

## Graphical Abstract

**Figure:**
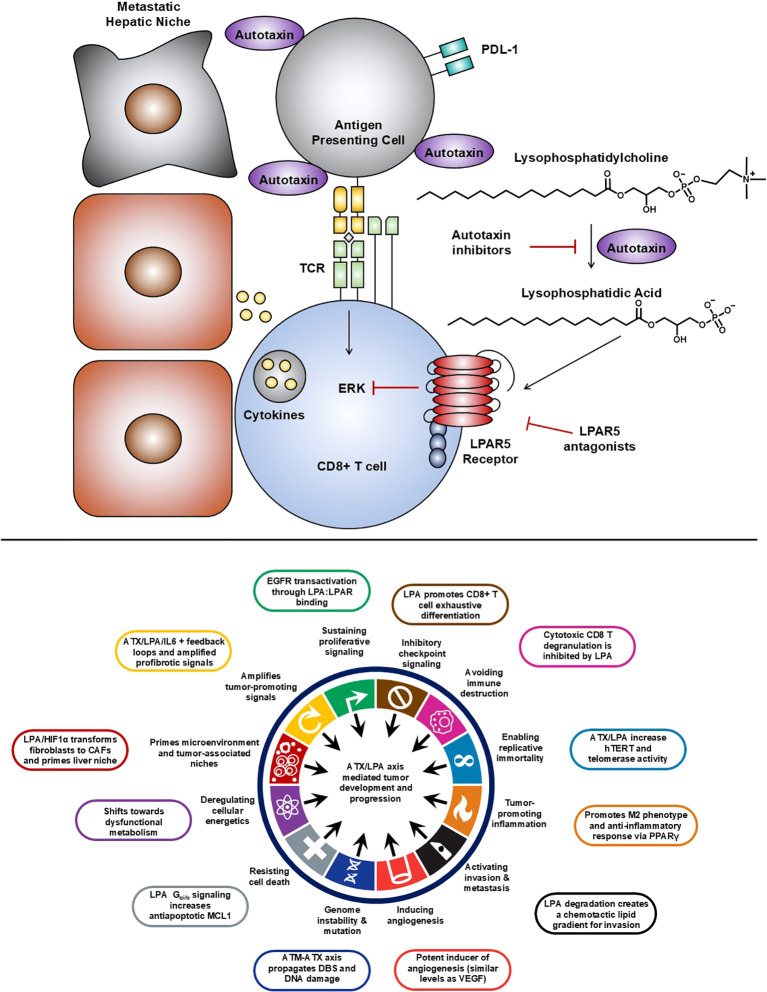
Autotaxin-lysophosphatidic acid signaling promotes hepatic Immune suppression in metastatic uveal melanoma, facilitates tumor development and progression, and may represent a therapeutic vulnerability for combination with HEPZATO.

## Introduction

Uveal melanoma is a rare subtype of melanoma that occurs within the orbit and has unique organotropism to the liver (1–3). Hepatic involvement occurs in up to 95% of uveal melanoma patients with stage IV disease and is associated with poor prognosis and high rates of mortality (4, 5). Patients with liver metastases typically present late, given the paucity of symptoms which often result in delayed diagnosis or treatment (2, 5, 6). Survival in patients with liver metastases without treatment ranges from 4–20 months (1, 4, 5). Despite advances in systemic immunotherapies that have transformed outcomes for cutaneous melanoma, uveal melanoma has remained refractory to most traditional and novel interventions (7). Checkpoint blockade with ipilimumab and nivolumab offers modest benefit for uveal melanoma patients (1, 7, 8). Reported response rates in uveal melanoma are 0–5% with anti–PD-1 monotherapy and 5–20% with combined ipilimumab and nivolumab (7, 9). Newer agents such as tebentafusp are limited to patients with HLA-A*02:01 positivity which excludes a significant proportion of individuals (1–3, 5, 10, 11). Unlike cutaneous melanoma, uveal tumors have a low tumor mutational burden and few structural genomic alterations, resulting in limited pharmacologic targets (12–14). These challenges underscore the need for novel therapeutic approaches to treat uveal melanoma.

Previous approaches to treat liver metastases in uveal melanoma, including surgical resection, trans-arterial chemoembolization, and Y-90 radioembolization, have modest objective response rates and brief progression-free survival and only limited extension in overall survival (1, 3, 5, 13, 15). There are newer treatment approaches for liver-directed therapy in uveal melanoma, including isolated liver perfusion techniques (16–19). HEPZATO is a type of percutaneous hepatic perfusion to deliver high localized concentrations of melphalan (16–19). This therapy was recently approved by FDA for uveal melanoma patient with hepatic dominant metastatic disease (e.g. <50% of liver involvement) in August 2023 (8, 17, 20). This type of liver-directed therapy is available to patients who are refractory to other treatments and/or are HLA-A*02:01 negative (8, 17, 19, 20). Since the recent implementation of HEPZATO, there are a handful of studies evaluating its effect and toxicity concluding it offers a regionalized approach for tumor control in the liver; it has no effect on extrahepatic metastases (16–19).

The liver is an immunotolerant organ due to constant exposure to gut-derived antigens, metabolites, and xenobiotics. Thus, to prevent pathologic hepatic inflammation there is a necessity to tightly regulate immune suppression (6, 21–23). This tolerogenic state is maintained by specialized hepatic antigen-presenting cells, including Kupffer cells and liver sinusoidal endothelial cells, which promote T-cell suppression through inhibitory checkpoint signaling, metabolic restriction, and immunosuppressive cytokine and lipid mediators (6, 21–23). Tumor cells exploit these intrinsic features of hepatic immune regulation to evade immune surveillance and establish metastatic niche (13, 24). In uveal melanoma, the liver is a predominant site of exclusive metastasis and impaired CD8^+^ T cell immunosurveillance is a central driver of disease progression and poor clinical outcomes (3, 6, 8, 13, 23, 24). The hepatic metastatic niche is reinforced by soluble liver-derived mediators including inhibitory proteins and bioactive lipid signaling pathways that suppress cytotoxic T cell function and promote dysfunctional and exhausted immune states (6, 13, 24–26). Autotaxin (ATX) is a secreted ecto-enzyme that is produced by hepatocytes in the liver and generates the immunosuppressive lipid, lysophosphatidic acid (LPA) (25, 27–29). Our laboratory has previously identified the ATX-LPA axis as an enzyme-lipid signaling mechanism that signals via LPAR5 to suppress cytotoxic CD8^+^ T cell function and promote dysfunctional and exhaustive signaling (26, 30–32).

ATX-generated LPA subsequently signals via LPA G-protein coupled receptors (LPARs) 1–6 expressed by diverse cell types (33). Expression of ATX and LPAR5 is cell and tissue-specific and controlled throughout the body (25, 26, 31). Physiologically within the liver, ATX is constitutively expressed by hepatocytes and LPAR5 expression is largely restricted to lymphocytes (27–29). ATX-LPA levels are increased locally in a microenvironment during normal signaling (25, 29). Under conditions of chronic inflammation such as cancer or chronic infection, ATX-LPA levels can be systemically elevated, and these changes are detectable in circulating blood (27, 29, 31). Previously, we have shown the ATX-LPA signaling on CD8 T cells subverts cytotoxic degranulation, impairs antitumor immunity and promotes exhaustion phenotypes in cancer and chronic infection (26, 30–32).

Here, we investigated clinical and preclinical therapeutic approaches to target metastatic liver disease in uveal melanoma. Specifically, we identify the ATX-LPA axis as a promising potential therapeutic target for uveal melanoma. We show ATX-LPA axis physiologically operates in the liver and find aberrant ATX expression by liver-derived CD8^+^ T cells from a uveal melanoma patient. We further show this ATX-LPA lipid signaling axis displays distinct signaling in CD8^+^ T cells. In effort to investigate potential new therapies, we go on to present a case of refractory uveal melanoma with liver-restricted disease being treated with HEPZATO, highlighting it as emerging therapeutic option for stabilizing liver disease. Our findings underscore the importance of exploring both established and emerging liver-directed strategies for uveal melanoma. We evaluate the liver microenvironment and implicate ATX-LPA as a contributor to local immune suppression. By linking clinically relevant hepatic therapy with immune signaling dysregulation, our study provides a framework for evolving treatment strategies for uveal melanoma.

## Materials and methods

### Clinical data review

All human research was approved by the Colorado and Mayo Clinic Institutional Review Board (IRB# 05-0309, #25-010199). A retrospective review of overall survival (OS) from time of diagnosis versus time from initiating immunotherapy was conducted on 37 uveal melanoma patients treated at the University of Colorado Anschutz Medical Campus. Additional retrospective case review was done on a patient with metastatic uveal melanoma treated with HEPZATO at Mayo Clinic, Rochester. Data were extracted from the electronic medical record, including demographics, comorbidities, disease history, prior systemic therapies (e.g., ipilimumab, nivolumab), HLA typing, and treatment-related toxicities. HEPZATO exposure was characterized by the number and timing of cycles, dosing, dose modifications, supportive measures, and adverse events. Tumor response was assessed radiographically using the Response Evaluation Criteria in Solid Tumors (RECIST) 1.1 criteria for hepatic and extrahepatic lesions at baseline, on-treatment, and post-treatment. Laboratory values, vital signs, and safety outcomes were collected longitudinally. Immunohistochemical, molecular, and patient-reported outcomes were included when available. Time-to-event descriptors, including duration from treatment initiation to first radiographic or clinical response, were calculated. Continuous variables (e.g., lesion diameters, laboratory values) were analyzed over time and relative to baseline. Data were visualized using GraphPad software.

### Mice and animal husbandry

All animal experiments complied with Institutional Animal Care and Use Committee (IACUC) regulations. Spleens were harvested from OT-I (CD45.2) mice gifted from Dr. Ross Kedl. Vα2 and Vβ5 transgenes were validated by mRNA and flow cytometrically. Both OT-I and *Lpar5^-/-^* OT-I mice were genotyped, bred, and maintained at the University of Colorado Anschutz School of Medicine. *Lpar5^-/-^* knockout was validated using quantitative real-time PCR. All animals were maintained in pathogen-free condition and housed within the regulation of the Institutional Animal Care and Use Committee.

### *In vitro* generation of effector CD8^+^ T cells

Effector CD8 T cells were generated from OT-I splenocytes which were homogenized. 0.83% NH_4_Cl-Tris Buffer was used to lyse red blood cells. OT-I splenocytes were cultured as a single cell suspension and peptide-pulsed with SIINFEKL (N4) at 2 µg/mL for 3 days at 37 °C. After day 3, the media was replaced with IL-2 (200-02, Peprotech, 1000 units/ml). Cells were then cultured for another 3 days. On day 7, effector CD8 T cells were collected at the middle interface layer from a Ficoll gradient. Polyclonal effector CD8^+^ T cells were generated using a similar *ex vivo* culture system but activated using anti-CD3/CD28 instead of N4. Effector CD8^+^ CD44^+^ T cells were determined to comprise >93% of the isolated culture. Antibodies used are as follows: CD8 BV421 (53-6.7, Biolegend), CD44 (103018, Biolegend), Vα2 (MR9-4, Biolegend), Vβ5 (B20.1, Biolegend). In some experiments, CD8 T cells were stimulated *in vitro* with 1ml of 5 µg/ml of anti-CD3 or 50 ng/mL of phorbol 12-myristate 13-acetate (PMA, Sigma-Aldrich #P8139) and 500 ng/mL of ionomycin (Sigma-Aldrich, #I9657). Cells were incubated with stimulation for 6 hours at 37 °C before flow cytometric processing and analysis.

### Liver harvest

Whole livers were excised from 8–10-week-old B6 mice and cut into ~1 mm pieces. Tissue was digested in Collagenase IV (5,000 U/mL in CLICKS/Eagle Hanks’ Amino Acids medium) at 37 °C for 30 min with intermittent mixing. The digested liver was filtered through a 100 µm strainer and washed with isolation buffer with Hanks’ Balanced Salt Solution containing 4.8% Bovine Serum Albumin (BSA) and 2 mM ethylenediaminetetraacetic acid (EDTA). Cells were centrifuged at 400×g for 5 min, resuspended in OptiPrep (20% in Phosphate Buffered Saline or PBS), and layered beneath PBS for density gradient centrifugation at 300×g for 15 min without brake. The interface layer was collected, filtered, and washed with isolation buffer, then resuspended in 2% FCS in PBS for downstream applications. For staining, cells were blocked with Fc receptor antibody (2.4G2) and incubated with surface or intracellular antibodies on ice for 20 min in the dark. Viability was assessed using Ghost Red 780 dye, and intracellular cytokines were detected following permeabilization with the Ebioscience™ Foxp3/Transcription Factor Staining Buffer Set (Thermo Fisher). After washing, cells were fixed in 1% paraformaldehyde (PFA) and stored at 4 °C until analysis by flow cytometry. Viable cells after collagenase treatment were quantified and normalized across samples prior to flow cytometric analysis. This protocol was performed similarly as previously detailed (34) and optimized for macrophage and Kupffer Cell isolation.

### LPA preparation and treatments

18:1 LPA (1-oleoyl-2-hydroxy-sn-glycero-3-phosphate; Avanti Polar Lipids) was stored in aliquots at −20 °C. Before use, aliquots were prepared at 1 mM in glutamine-supplemented RPMI (Roswell Park Memorial Institute medium) and sonicated for 30 minutes to ensure complete dispersion.

### Quantitative real time PCR

Either mouse splenocytes or human CD8^+^ T cells were used for quantitative real time polymerase chain reaction (PCR). Splenocytes were isolated into single cell suspensions as described above. Human peripheral blood mononuclear cells (PBMCs) were collected from peripheral blood. The study was approved by the Colorado Institutional Review Board (IRB# 05-0309) and conducted in accordance with the Declaration of Helsinki. The participant provided written informed consent. PBMCs were run over a Ficoll gradient, and bead-based CD8 T cell isolations were performed (130-396-495, Miltenyi Biotec). Cells were homogenized and subjected to on-column DNase I digestion (Qiagen) to eliminate DNA contamination. RNA was reverse-transcribed with the Verso cDNA Synthesis Kit (Thermo Fisher Scientific), and qPCR analyses were conducted in triplicate using PowerUp SYBR Green Master Mix (Thermo Fisher Scientific) on a StepOne Plus real-time PCR system (Applied Biosystems). *ENPP2* primers sequences are as follows, sense: 5’ ACAACGAGGAGAGCTGCAAT 3’ and anti-sense: 5’ GAAGTCCAGGCTGGTGAGA 3’. *Enpp2* primers sequences are as follows, sense: 5’ GCCCTGATGTCCGTGTATCT 3’ and anti-sense: 5’ CGTTTGAAGGCAGGGTACAT 3’. *PDCD1* primer sequences are as follows, sense: 5’ CAGAGCTCGTGGTAACAGAG 3’ and anti-sense: 5’ CAATGACCATGCCTTGAAACC 3’.

### Western immunoblotting

Cell pellets containing a minimum of 2 × 10^6 cells were lysed in ice-cold RIPA buffer supplemented with a protease and phosphatase inhibitor cocktail (Thermo Fisher Scientific). Protein concentrations were determined using a Bradford assay, and 50 µg of protein per sample was resolved by SDS-PAGE. Proteins were transferred to nitrocellulose membranes using a dry transfer system. Immunoblotting was performed with the following primary antibodies: phospho-MEK1/2 (Ser217/221, #9121, Cell Signaling Technology), MEK1/2 (#9122, Cell Signaling Technology), phospho-ERK1/2 (Thr202/Tyr204, #4370, Cell Signaling Technology), ERK1/2 (#9102, Cell Signaling Technology), phospho-PLCγ (Tyr783, #D6M9S, Cell Signaling Technology), PLCγ (#5690S, Cell Signaling Technology), PD-1 (#D4W2J, Cell Signaling Technology), and β-actin (#4970, Cell Signaling Technology).

### Flow cytometry

Cells were stained in MACS (magnetic-activated cell sorting) buffer (PBS containing 2% Fetal Calf Serum and EDTA). Fc receptors were blocked with 2.4G2 antibody (70-01610-M001, Invitrogen) during a 20-minute incubation on ice. Viability was assessed using Ghost Red 780 dye (13-0865-T100, Tonbo Biosciences). For intracellular cytokine analysis, lymphocytes were plated with 3 µg/mL of Brefeldin A (Sigma-Aldrich) and cells were permeabilized and stained using the Ebioscience™ Foxp3/Transcription Factor Staining Buffer Set (00-5523-00, Thermo Fisher) using MACS buffer (PBS with 0.5% BSA and 2mM EDTA). Flow cytometry was performed on CytoFlex or LSRII instruments (BD), and data were analyzed with FlowJo v8 (TreeStar). Antibodies employed included CD8 BV421 (100753, BioLegend), CD8 PerCP (100731, BioLegend), CD44 Alexa Fluor 647 (103018, BioLegend), CD44 Alexa Fluor 700 (103025, BioLegend), CD3 BV421 (100335, BioLegend), CD69 FITC (104505, BioLegend), CD107a Alexa Fluor 488 (121608, BioLegend), IL-2 PE (503808, BioLegend), TNFα PE-Cy7 (506324, BioLegend), and IFNγ APC (505809, BioLegend). CD45 Pacific Blue (103125, Biolegend), CD14 APC (12311, Biolegend), CD11b FITC (101205, Biolegend), F/480 Alexa Fluor 700 (123129, Biolegend), PD-L1 (124331, Biolegend), ATX (clone 7A5, MABT1350-100UG, Sigma-Aldrich).

### Schematic figures

Schematic figures were created with BioRender.com or self-generated by the authors.

## Results

### Exploring ATX-LPA as a potential therapeutic target in uveal melanoma

**Figure 1:**
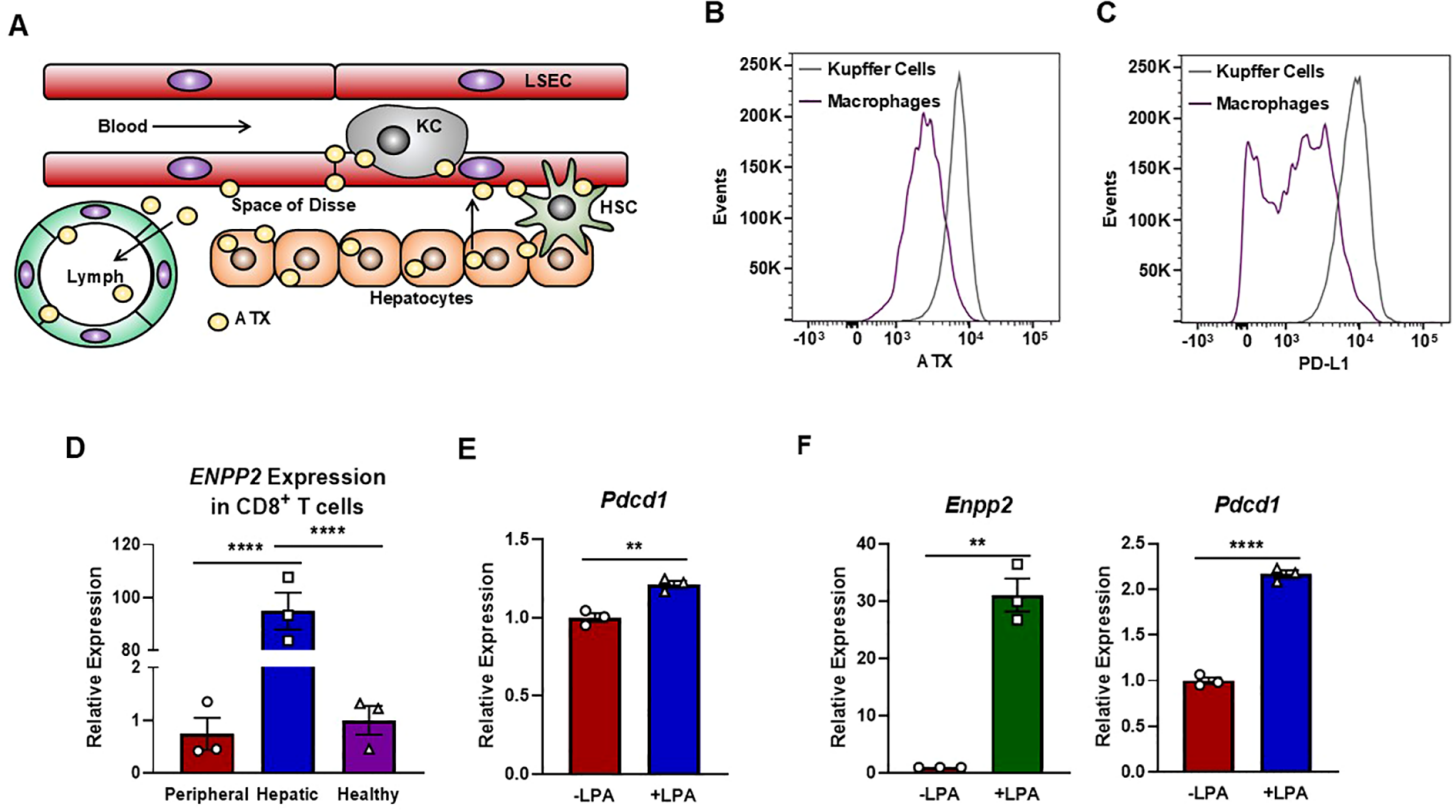
Autotaxin is elevated within the hepatic niche. **(A)** Schematic representation of the secreted enzyme autotaxin (ATX, yellow) which can adhere to local hepatocytes (orange), Kupffer cells (KCs, grey), or hepatic stellate cells (green). In large enough quantities, ATX can disseminate from the Space of Disse into the lymphatic vessels (green and purple) to elevate systemic levels of ATX. **(B, C)** Flow cytometric histograms displaying expression of **(B)** ATX or **(C)** PD-L1 on Kupffer Cells (grey) or macrophages (purple). Panels are representative histograms from one experiment which was replicated in two independent experiments (two biological replicates) with similar results. **(D)** Quantitative real time PCR (qrtPCR) expression of *ENPP2* (the gene name for ATX) from CD8^+^ T cells isolated either from peripheral blood (red), liver (blue), or healthy peripheral blood (purple). Data represent three biological replicates with a one-way ANOVA that was performed with multiple comparisons with **** p<0.0001. **(E, F)** qrtPCR expression of *Pdcd1* and *Enpp2* from **(E)** antigen specific or **(F)** polyclonal effector CD8^+^ T cells treated *ex vivo* with or without lysophosphatidic acid (red indicates without LPA supplementation; blue indicates LPA supplementation). Both panels E and F represent samples from 3 biological replicates. Statistics were performed using an unpaired Student's t test was preformed with **p<0.005.

Within the hepatic niche, ATX is normally produced by resident cells and associates on the surface of cells including Kupffer cells (KCs, liver-specific macrophages) (**Figure 1A**) and can be detectable flow cytometrically on KCs (27–29). We show that ATX is expressed in the liver of healthy B6 mice where it can be found on the surface of KCs isolated from whole liver harvest, demonstrating hepatic localization (**Figure 1B**; **Supplementary Figure 1**). Furthermore, ATX-associated KCs display increased expression of PD-L1 (**Figure 1C**; **Supplementary Figure 1**) as opposed to non-resident macrophages supporting a role for an ATX-LPA axis contributing to an immunosuppressive microenvironment under normal conditions. Since tumor cells frequently exploit the ATX-LPA axis, we measured ATX expression by liver-derived CD8^+^ T cells isolated from a uveal melanoma patient with hepatic metastases and found significantly increased transcript expression compared to CD8^+^ T cells in peripheral blood from the same patient or a healthy donor (**Figure 1D**). We also determined that exogenous LPA increases *PD-1* expression on murine effector CD8^+^ T cells (**Figure 1E**). We further confirmed these findings on polyclonal activated CD8^+^ T cells murine models (**Figure 1F**). Our previous work demonstrated LPA signaling increases exhaustive differentiation with upregulation of PD1, Lag3, and Tox on antigen-specific CD8^+^ T cells (31). Collectively, these findings suggest that the ATX-LPA axis is elevated in hepatic cells and may play a role in liver-mediated immunosuppression via PD-L1/PD-1 induction.

### Distinct MAPK signaling dynamics in ATX-LPA vs TCR-mediated signaling in CD8^+^ T cells

**Figure 2:**
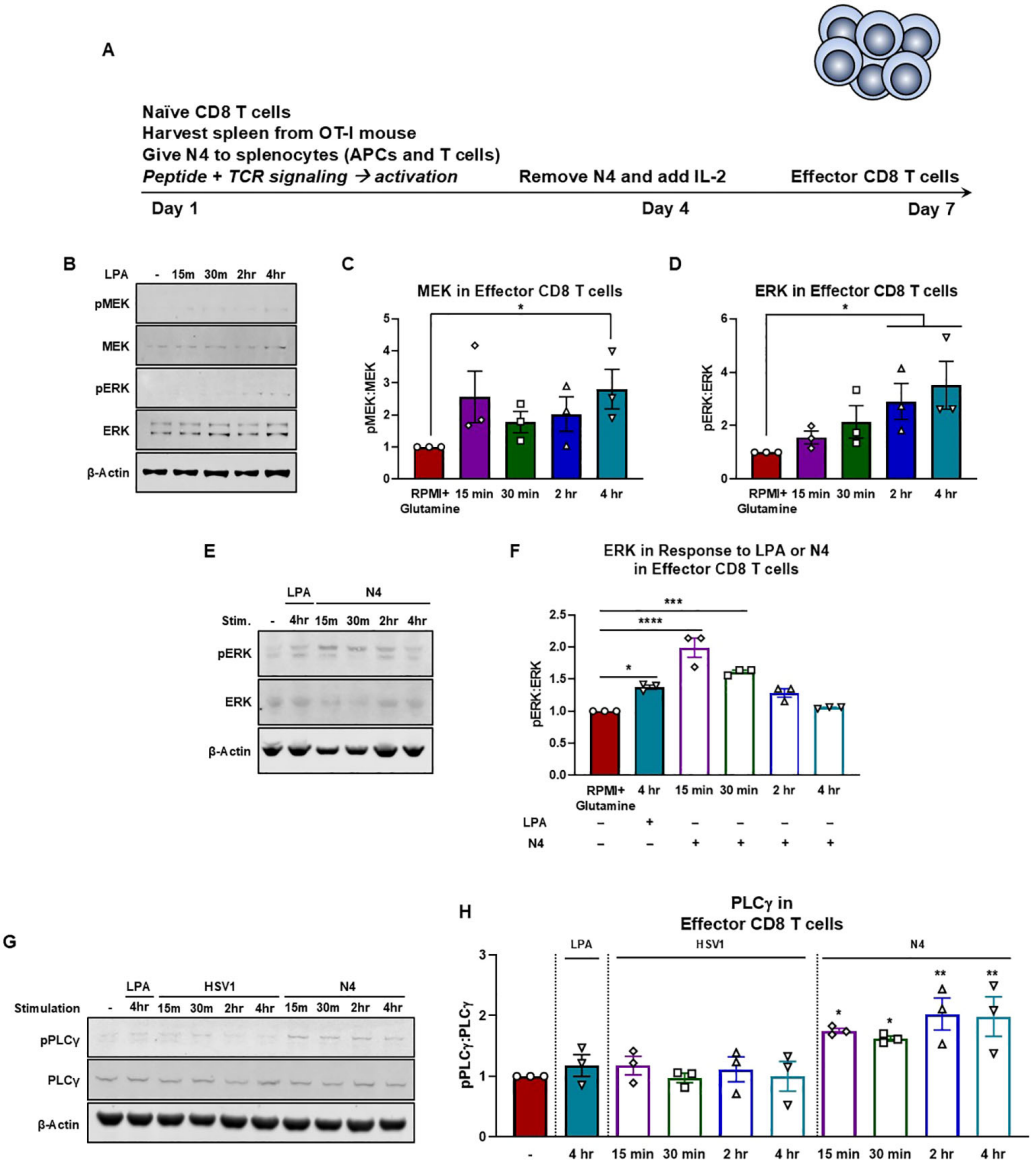
Lysophosphatidic acid activates the mitogen-activated protein kinase pathway at low levels. **(A)** Schematic for generating effector CD8 T cells *ex vivo* where antigen presenting cells (APCs) are pulsed with SIINFEKL (N4). Cells are kept in culture and using IL-2 until day 7. **(B)** Western immunoblot of phosphorylated MEK and ERK on OT-I CD8 T cells treated with 1 µM of lysophosphatidic acid for 15 minutes (15m), 30 minutes (30m), 2 hours (2hr), or 4 hours (4hr). Data represent n = 3 mice, and experiments were performed as three independent experiments (e.g. three biological replicates). **(C, D)** Quantification of phosphorylated:total protein for **(C)** MEK and **(D)** ERK. **(E)** Western immunoblot of phosphorylated ERK on OT-I CD8 T cells peptide pulsed with N4 or lysophosphatidic acid for 15 minutes, 30 minutes, 2 hours, or 4 hours. Data represent n = 3 mice, and experiments were performed as three independent experiments (e.g. three biological replicates). **(F)** Quantification of panel **(E)** measuring phosphorylated ERK (pERK):total ERK. (n = 3 mice, and experiments were performed as three independent experiments). **(G)** Western immunoblot of effector CD8 T cells pulsed with either LPA, HSV1 (SSIEFARL), or N4 (SIINFEKL) for either 15 minutes, 30 minutes, 2 hours, or 4 hours. Data represent n = 3 mice, and experiments were performed as three independent experiments (e.g. three biological replicates). **(H)** Quantification of panel **(G)** representing the amount of phosphorylated PLCγ (pPLCγ):total PLCγ. Statistics for the entire figure were performed using an ANOVA with a Tukey’s post-hoc analysis * p < 0.05, ** p < 0.005, *** p < 0.0005, **** p <0.0001.

To further examine the role of ATX-LPA axis in immunosuppression, we determined whether LPA signaling by CD8^+^ T cells affects downstream T cell antigen receptor (TCR) signaling. We have previously demonstrated that LPAR signaling in the context of acute TCR stimulation impairs calcium release downstream of PLCγ and the IP_3_R. Here, we evaluated whether LPA signaling also influences TCR-mediated induction of the MAPK pathway, a potential contributor to dysfunctional immune signaling. Using antigen-specific cytotoxic effector CD8^+^ T cells generated *ex vivo* from OT-I mice, we assessed MAPK signaling with LPA treatment by western immunoblotting (**Figure 2A**). Previously studies have shown that TCR-mediated MAPK signaling can peak within minutes (35–37) and recent data further demonstrate that antigens with high TCR affinity promote sustained peak ERK signaling for approximately 20 minutes depending on cell type (38). Based on these experiments we selected time points between 15 minutes and 4 hours based on previously established temporal dynamics of LPA signaling (31). First, we observed that LPA alone (e.g. in the absence of TCR stimulation) induced a modest but persistent MEK and ERK phosphorylation, which became significant by 4 hours (**Figures 2B–D**; **Supplementary Figure 2**). In contrast, TCR stimulation with SIINFEKL (also termed N4) triggered rapid, transient ERK phosphorylation that peaked at 15 minutes and by 4 hours post-treatment had returned to baseline and was not significantly different than TCR-induced phosphorylated ERK (**Figures 2E, F**; **Supplementary Figure 3**). These data show LPA and TCR signals converge on MAPK components but display distinct temporal kinetics. TCR-mediated MAPK signaling resulted in more rapid “on-off” signal, whereas LPA alone caused low-level ERK phosphorylation that persists at 4 hours.

### LPA does not affect TCR-mediated MAPK signaling

Since the ATX/LPA axis signaled via the MAPK pathway in CD8^+^ T cells similar to the TCR, but with an important kinetic distinction, we next assessed potential cross-talk between LPAR and TCR activation signaling pathways. Using western immunoblotting, we first evaluated downstream PLCγ activity, measured by phosphorylated PLCγ, in effector CD8^+^ T cells stimulated with LPA, N4, or an irrelevant negative control peptide HSV1 (SSIEFARL). We found that LPA did not promote PLCγ activation (**Figures 2G, H**; **Supplementary Figure 4**) and was distinct from the canonical TCR signaling pathway. Therefore, LPAR signaling increases MAPK phosphorylation but does not rely on the PLCγ aspect of TCR signaling, highlighting key differences between LPAR and TCR signaling in terms of temporal MAPK dynamics and other signaling mechanisms.

**Figure 3:**
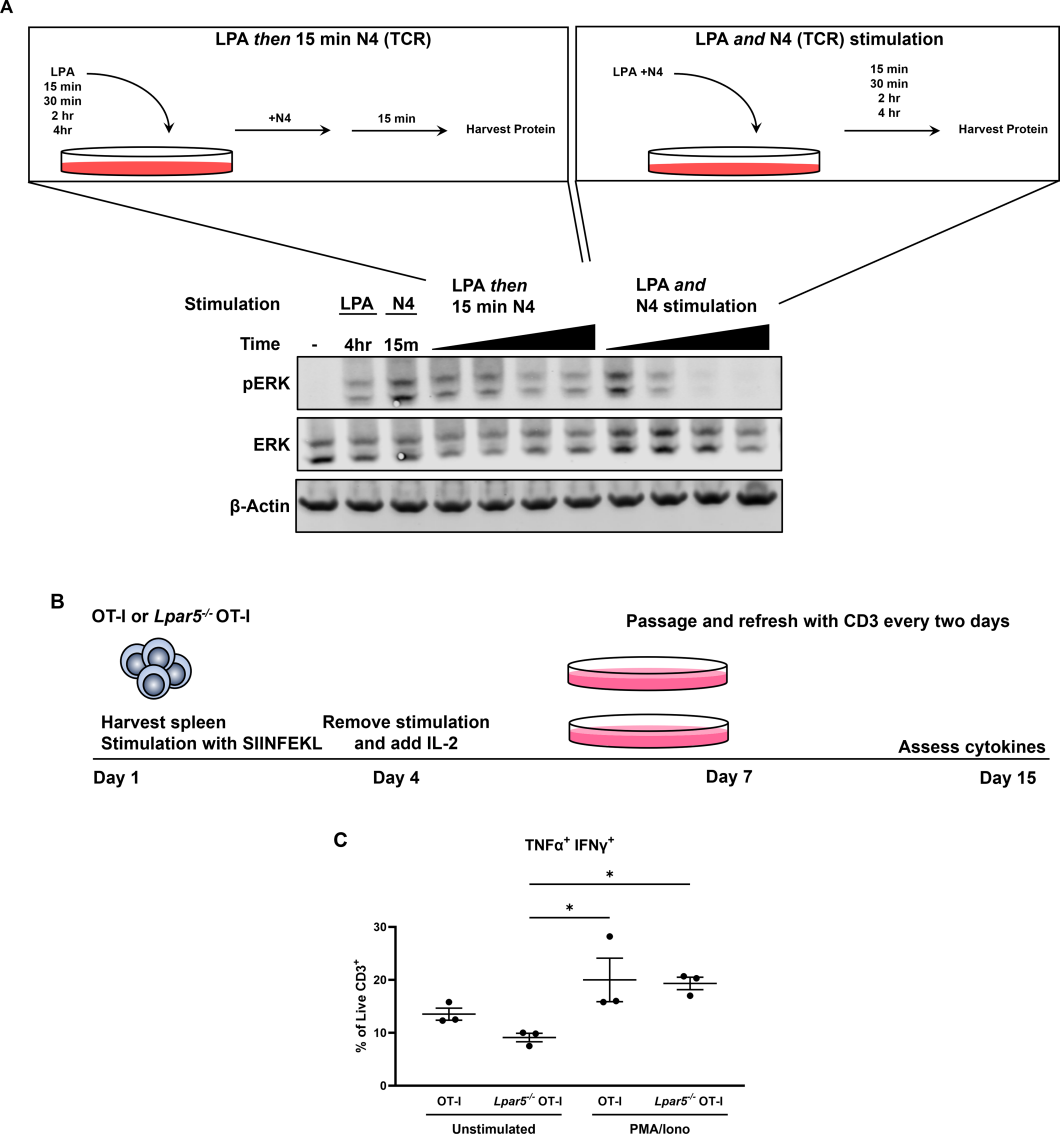
Lysophosphatidic acid suppresses basal ERK signaling but fails to override TCR-driven ERK activation. **(A)** Experimental design schematic of lysophosphatidic acid (LPA) administered 15 minutes, 30 minutes, 2 hours, or 4 hours before SIINFEKL (N4) stimulation and LPA and N4 co-administered for 15 minutes, 30 minutes, 2 hours, or 4 hours. Below is the western immunoblot of phosphorylated ERK and total ERK proteins in effector CD8 T cells. This experiment was repeated in two separate and independent biological replicates with similar results. **(B)** Schematic of experimental design for *in vitro* cytokine stimulation. **(C)** Percent of viable CD3^+^ cells triple-producing TNFα, IFNγ in wildtype OT-I or *Lpar5*^-/-^ OT-I T cells chronically stimulated with CD3. Data represent n = 3 mice, and experiments were performed as three independent experiments (e.g. three biological replicates). In these experiments, baseline unstimulated conditions did not demonstrate a consistent reduction in CD8+ T cell frequencies in *Lpar5*^-/-^ OT-I cells compared to wild-type OT-I controls. Statistics for this experiment were performed using a two-way ANOVA * p < 0.05.

We have previously determined that LPA signaling impairs subsequent TCR granule exocytosis and cytotoxicity (26, 31, 32). Yet, it has remained unclear whether LPA induces a prior signal that alters the subsequent ERK response to TCR engagement (e.g. attenuates subsequent ERK signaling) or competes with TCR signaling. Using our *in vitro* model and western immunoblotting, we assessed ERK phosphorylation with either pre- or co-treatment of LPA during TCR signaling (**Figure 3A**; **Supplementary Figure 5**). We observed that LPA, when given as a pre-existing signal, resulted in persistent ERK signaling with low level ERK phosphorylation at 4 hours. Interestingly, co-administration of LPA and N4 (and thus concurrent LPAR and TCR signaling) does not appear to blunt TCR-mediated ERK phosphorylation at 15 minutes and resolves by 4 hours similar to TCR signaling in the absence of LPA stimulation. These data argue that LPA does not appear to regulate downstream TCR-induced ERK signaling.

### The ATX-LPA axis represses CD8^+^ T cell cytokine production

Dysfunctional signaling typically precedes impaired CD8 T cell activity (39). As we observed LPA did not influence downstream TCR-mediated ERK signaling, we assessed whether LPA altered CD8 T cell functional activity as measured by cytokine production in extended *in vitro* cultures of effector cytotoxic CD8 T cells. In these experiments we performed flow cytometric cytokine analysis after *ex vivo* treatment of wild-type and LPAR5-deficient (*Lpar5^-/-^*) effector OT-I CD8^+^ T after persistent TCR stimulation over 15 days using N4-mediated antigen activation and subsequent anti-CD3 restimulation (**Figure 3B**). These results demonstrated that splenocytes from *Lpar5^-/-^* OT-I mice not only harbored a significantly increased frequency of live CD8^+^ cells relative to wild type (**Figure 3C**; **Supplementary Figure 6-7**). Altogether, these findings suggest that LPA signaling acts upstream of TCR and promotes tolerant CD8^+^ T cells.

### HEPZATO as an emerging non-immune-based option for uveal melanoma patients

**Figure 4:**
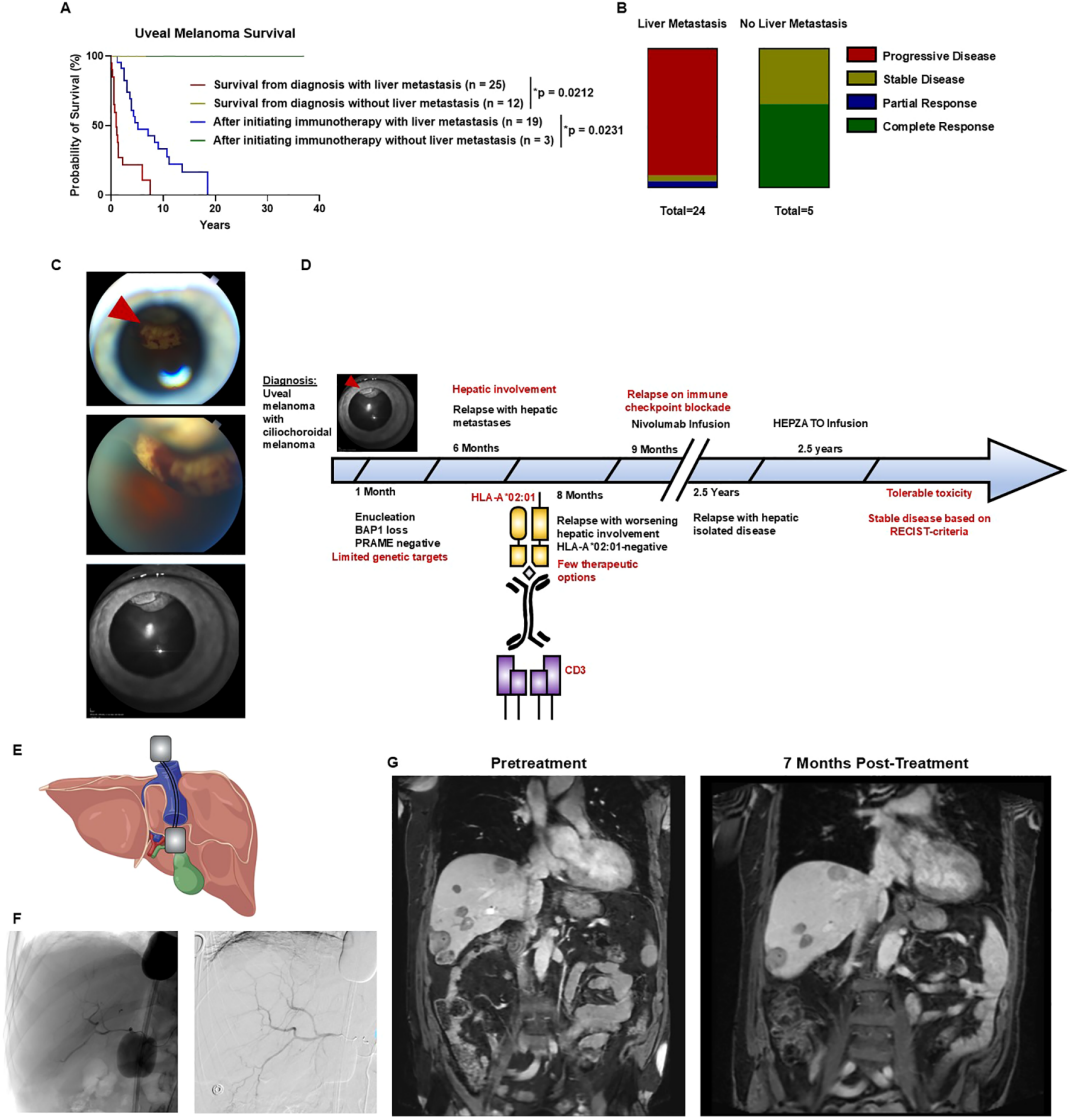
Hepatic metastasis drives poor outcomes in uveal melanoma but liver-directed therapies are promising therapies. **(A)** Overall survival in uveal melanoma with and without liver involvement either from the time of diagnosis (red and yellow) or after initiating immunotherapy (blue and green). **(B)** Stacked bar plots of patient response rates to immune checkpoint blockade. **(C-G)** Clinical case presentation and management of metastatic uveal melanoma. **(C)** Initial case presentation with ciliochoroidal uveal melanoma and **(D)** subsequent management of disease course. **(E)** Schematic of HEPZATO approach to introducing a double balloon catheter in the retro-hepatic inferior vena cava for isolated liver perfusion. **(F)** Radiographs of high dose melphalan perfusion. **(G)** Radiographic evidence of hepatic disease stabilization prior to HEPZATO and 7 months post-treatment.

Uveal melanoma patients have poor outcomes to current therapies, particularly those with liver metastases (1, 3–5, 7, 10, 11). We retrospectively analyzed outcomes in a patient cohort of 37 treated uveal melanoma patients and observed patients with hepatic metastases compared to those without hepatic metastases (e.g. distant extrahepatic metastases or locally advanced/recurrent disease), had significantly worse overall survival (**Figure 4A**). Even with the advent of newer immunotherapies, uveal melanoma patients with hepatic involvement almost uniformly experienced progressive disease while on therapy and had significantly worse survival than those without liver metastases (**Figures 4A, B**). Here, we present a case of a uveal melanoma patient treated with HEPZATO. A 55-year-old gentleman first presented with flashing lights and vision in loss in his left eye. Upon presentation to his local eye doctor, they found a superior, anterior uveal mass measuring 24.1 x 20.6 x 10.2 mm and was biopsied revealing a ciliochoroidal melanoma (**Figures 4C,D**). He underwent enucleation and was found to have limited actionable genetic targets. Six months after diagnosis, he relapsed with hepatic metastases and was noted to have worsening disease at an interval of 2 months. At that time, he was found to be negative for HLA-A*02:01, rendering him ineligible for the bispecific antibody, tebentafusp. He was subsequently started on anti-PD-1 immune checkpoint blockade with nivolumab. He remained stable on single agent nivolumab for more than 1.5 years until he experienced progression of hepatic metastases. He then proceeded with HEPZATO therapy. HEPZATPO introduces a double balloon catheter into the retro-hepatic inferior vena cava to perform isolated hepatic perfusion of high dose melphalan (**Figures 4E,F**). This approach achieves cytotoxic drug concentrations while limiting systemic exposure. At an interval of 7 months, the patient’s disease remained stable based on RECIST 1.1 criteria (**Figure 4G**). The patient has remained more than 9 months post HEPZATO infusion with liver disease stabilization and tolerable toxicity. This case exemplifies and corroborates the existing data that the liver-directed therapy, HEPZATO, can stabilize hepatic disease. Therapeutic efficacy may be limited by the immunosuppressive microenvironment of the liver, underscoring the need to understand how immune tolerance is maintained and altered within the hepatic niche to inform complementary immune-based approaches.

## Discussion

Our findings highlight that uveal melanoma patients with liver metastases have markedly worse outcomes, resulting in a critical need to improve, or develop new, therapies for these patients. Here, we discussed and reviewed current clinical and preclinical approaches to targeting uveal melanoma. Despite successes of immune-based treatments in cutaneous melanoma, uveal melanoma is generally not responsive to immunotherapy. We identify the ATX-LPA axis as a potential mechanism contributing to immunosuppression in uveal melanoma liver metastases. In the setting of hepatic metastasis in uveal melanoma, ATX transcript expression is significantly elevated in CD8^+^ T cells and could contribute to dysfunctional hepatic immune responses. This contrasts with T cells from healthy individuals or wild type mice, where ATX is not normally expressed under homeostatic settings. Our data is consistent with previously published literature conferring the liver as a physiologically immunotolerant organ (6, 21, 22).

The ATX-LPA axis is a potentially targetable signaling axis given that LPA signaling by T cells is mediated by G-protein coupled receptors. To date, there are clinical studies investigating ATX inhibitors in idiopathic pulmonary fibrosis (33, 40). Despite the development of ATX inhibitors, there is a clear need to better understand and develop new therapeutic strategies for targeting ATX-LPA mediated immune suppression in tumors. Here, we show that LPA signaling by CD8^+^ T cells proceeds via a mechanism(s) that differs from TCR-induced activation. We find that TCR signaling on CD8 T cells elicits a rapid “on-off” phosphorylation of ERK. Generally, immune cell effector functions are dependent on oscillatory signals (35, 38, 41). Importantly, the signaling differences observed in our study are unlikely to be explained solely by the potency of the activating stimuli but rather represents the integrated balance between activating and inhibitory networks. MAPK propagation is constrained by negative-feedback mechanisms, including ERK-dependent feedback loops, induction of dual-specific phosphatases (DUSPs), receptor desensitization, and other regulatory checkpoint programs (42–44). Such pathways can modulate the amplitude and temporal dynamics of downstream signaling, thereby influencing functional responses (42, 43). In this context, persistent low-level ERK phosphorylation induced by LPA could represent weak activation versus qualitatively distinct signal shaped by feedback inhibition that blunts subsequent TCR responsiveness. This concept is consistent with prior observations that chronic or dysregulated ERK signaling can accompany anergic or dysfunctional immune states (42, 45, 46). Future studies dissecting how LPA signaling interfaces with ERK feedback regulation and other inhibitory circuits may further clarify how the hepatic metastatic microenvironment enforces CD8^+^ T cell dysfunction. Additional studies should also be done on downstream MAPK components (p38 and JNK) should be evaluated in CD8^+^ T cell exhaustive programming in future studies.

Persistent low-level ERK signaling has also been observed in anergic immune cells and impaired immunity (46, 47). We demonstrate that LPA promotes persistent ERK signaling that promotes dysfunctional TCR signaling. In our studies, LPA does this as a preceding signal and is not inherently potent enough to overcome TCR-induced ERK signaling but can pre-program a dysfunctional response. Pre-treatment with LPA results in dynamic loss of oscillatory ERK signaling, and genetic ablation of *Lpar5* in mice results in improved cytokine release. This is to say, that LPA may pre-program immunosuppression. Typically, dysfunctional immune responses in cancer go hand in hand with exhaustive phenotyping (31, 39). We have previously shown that LPAR signaling promotes exhaustive phenotypes (29, 31). Our current understanding of exhaustion is rapidly evolving, and it is becoming increasingly important to recognize non-canonical mechanism of exhaustion and exhaustive-like programming. Additional studies should be performed to determine if exhaustion could be pre-programed by LPA signaling.

To date, our understanding of the mechanisms that govern organotrophic liver metastasis in uveal melanoma patients remains poor, and identifying the mechanisms leading to this phenomenon is critical to improving outcomes for these patients. Uveal melanoma patients most frequently acquire hepatic disease involvement with advanced stages (1–4, 10, 13, 14). The liver remains to be an immunotolerant microenvironment which likely shapes immune responses to metastasis (6, 21, 22). Patient-derived tissue and liver-derived CD8^+^ T cells were a limited resource and due to constraints in sample availability and cell yield, we were unable to replicate protein assessments or include multiple patient samples. Thus, the inherent tolerance of the liver combined with tumor-driven ATX-LPA signaling may create a permissive niche for metastatic progression.

Several other solid tumor malignancies are also not immunogenic or responsive to single agent immunotherapy and require the combination of chemotherapy and immunotherapy (20, 48). HEPZATO is a promising liver-directed chemotherapy and could potentially provide durable local disease control with tolerable toxicity in patients with otherwise limited options. To date, we have early clinical implementation of liver-directed therapy with HEPZATO as a type of isolated liver perfusion which can stabilize liver disease (16, 17, 19, 48). Thus, the introduction of HEPZATO for liver-dominant metastatic uveal melanoma presents a compelling opportunity to pair hepatic perfusion with a chemotoxic agent with other immunotherapies. Additionally, uveal melanoma having a poor response to immunotherapy may reflect that the liver is an immunosuppressive microenvironment and thus could benefit from a different type of immune-based therapy. Combining systemic inhibition with ATX-LPA blockade and HEPZATO could both debulk hepatic disease and potentially restore anti-tumor immunity. Beyond cytoreduction alone, regional melphalan perfusion may also have the potential to remodel the hepatic metastatic niche by reducing tumor burden and depleting local cellular sources of immunosuppressive mediators, including macrophage or tumor-derived ATX. Isolated hepatic delivery of high dose chemotherapy could increase tumor antigen release, improve antigen presentation and in combination with ATX-LPA pathway inhibition, could reduce immunosuppression in the hepatic metastatic niche. However, it remains unknown how high dose melphalan (or any other liver directed therapy) may impact the ATX-LPA axis. Although the direct effects of melphalan on ATX expression, LPA production, and LPA receptor signaling remain unknown, these mechanisms warrant future investigation in preclinical liver metastasis models. In the future, it could be that ATX-LPA axis is a vulnerability to exploit in the adjuvant or neoadjuvant setting alongside other treatments trialed for liver isolated disease in uveal melanoma. Specifically, liver transplant has been studied in metastatic colorectal cancer with isolated liver disease (49). This type of therapy has yet to be examined in uveal melanoma but could also become another therapeutic option for uveal melanoma patients in the future.

The ATX-LPA pathway could be targeted via direct ATX inhibition to reduce the local and systemically circulating levels of LPA. Alternatively, another compelling therapeutic target is the LPAR5 receptor which is more selectively expressed on lymphocytes. In the setting of uveal melanoma liver metastasis, where both hepatic immune tolerance and elevated ATX-LPA signaling may coexist, LPAR5 provides a mechanistically specific checkpoint distinct from canonical PD-1/CTLA-4 pathways. Targeting LPAR5 therefore offers the potential to restore CD8^+^ T cell effector fitness, enhance cytokine production, and improve immune-mediated tumor control without requiring direct tumor antigen targeting. Direct ATX inhibition would have similar downstream effects on CD8^+^ T cells but also the available levels of LPA signaling onto the tumor cells, non-parenchymal resident cells, and other circulating immune cells. Both ATX inhibition and LPAR5 targeting are both pharmacologically tractable and may be amendable to small-molecule targeting or combination strategies with checkpoint blockade or liver-directed therapies such as HEPZATO.

Altogether, our findings underscore the clinical challenges of treating uveal melanoma with liver metastases. The standard of care in cutaneous melanoma is immunotherapy which provides only limited benefit in uveal melanoma (3, 7, 14, 15). Yet the immunosuppressive hepatic niche remains to be a major barrier to durable therapeutic responses in uveal melanoma. We identify the ATX-LPA axis as a possible mechanism for hepatic immune evasion. ATX-LPA promotes dysfunctional CD8^+^ T cell phenotypes and could be a potential preclinical therapy. We also highlight HEPZATO as a potential therapeutic for uveal melanoma immunotherapy combination. In summary, the treatment of uveal melanoma will continue to evolve across the preclinical to clinical spectrum with more translational avenues to overcome hepatic metastasis and immune tolerance.

## Conclusion

Metastatic uveal melanoma remains a challenging disease with unique organ tropism to the liver where there is intrinsic immune tolerance and limited responsiveness to conventional immunotherapy. In this study, we identify the ATX-LPA axis as a plausible mediator of hepatic immune suppression and CD8^+^ T cell dysfunction in the liver metastatic niche. Our findings demonstrate that LPA signaling induces aberrant, persistent ERK activation distinct from canonical T cell receptor signaling and that disruption of the LPA receptor signaling restores CD8^+^ T cell function under chronic stimulation. These data support the ATX-LPA pathway as a rationale immunologic target in uveal melanoma.

We further highlight the clinical relevance and application of liver-directed therapies through HEPZATO, which may provide meaningful hepatic disease control in select patients with liver-dominant metastatic disease. Together, these observations support a translational framework in which regional cytoreductive therapies could be combined to overcome hepatic immune suppression. Future studies should define how liver-directed treatments alter the metastatic microenvironment and evaluate whether ATX-LPA pathway inhibition could synergize with immunotherapy or hepatic perfusion of chemotherapy. Advancing such therapies may be essential to improve outcomes for patients with metastatic uveal melanoma.

## Data Availability

The original contributions presented in the study are included in the article/**Supplementary Material**. Further inquiries can be directed to the corresponding authors.
