# Supplementary figures and images for: Targeting liver metastases in uveal melanoma: ATX-LPA mediated immunosuppression and novel therapeutic approaches
Source: Front Immunol. 2026 Jun 18;17:1829486. doi: 10.3389/fimmu.2026.1829486 (PMC13322911; doi:10.3389/fimmu.2026.1829486)

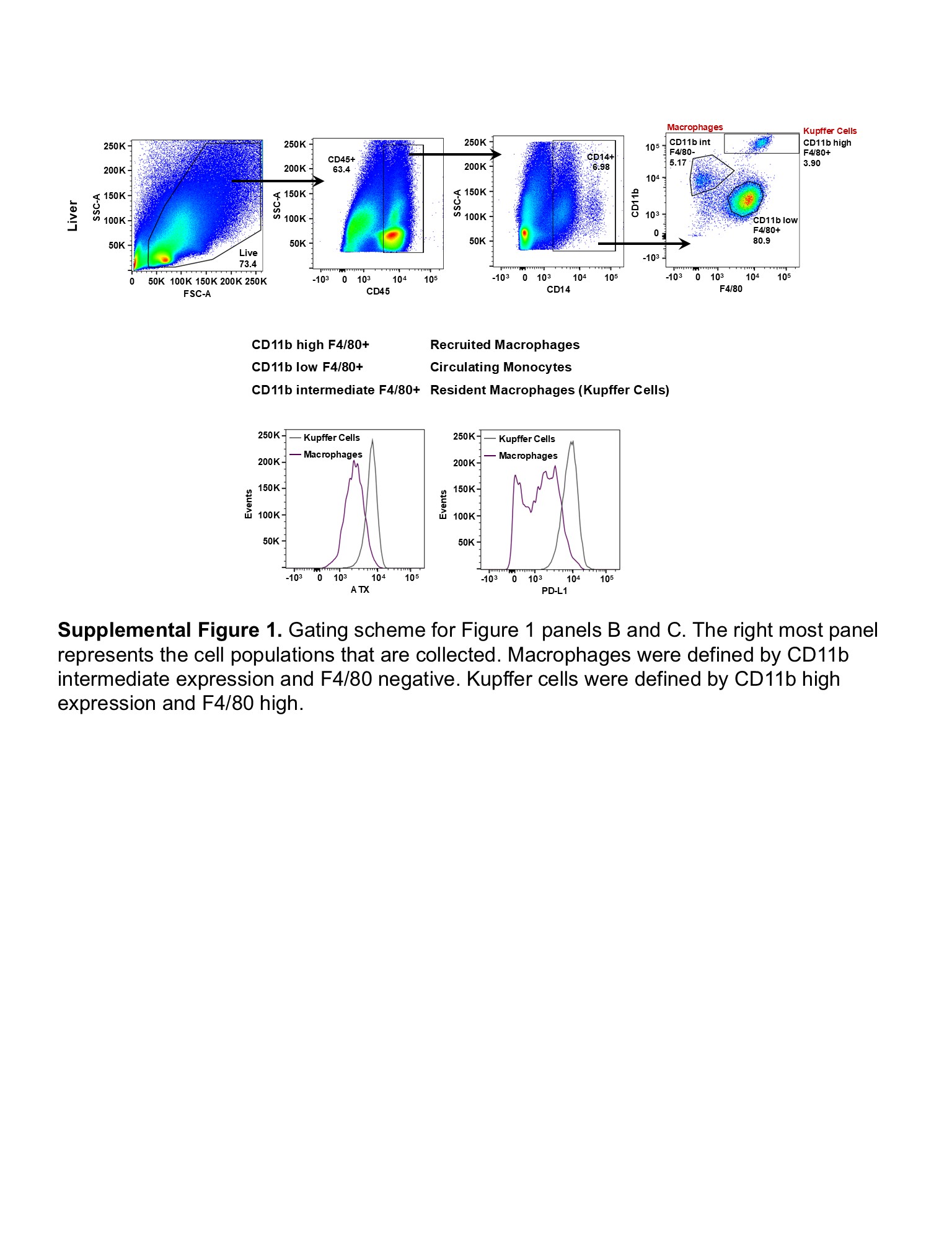

Supplement: Supplementary file 1 [file Image1.jpeg]

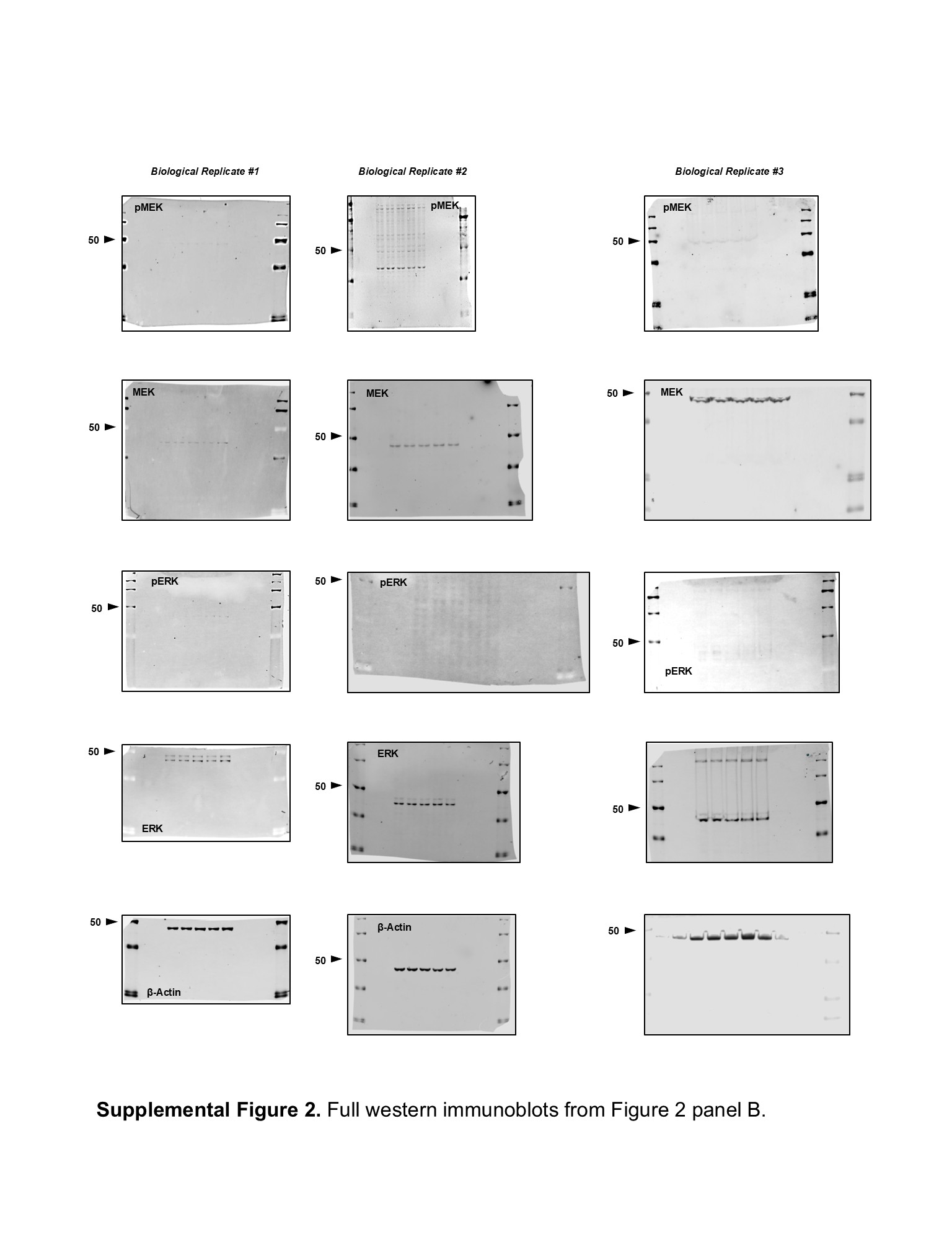

Supplement: Supplementary file 2 [file Image2.jpeg]

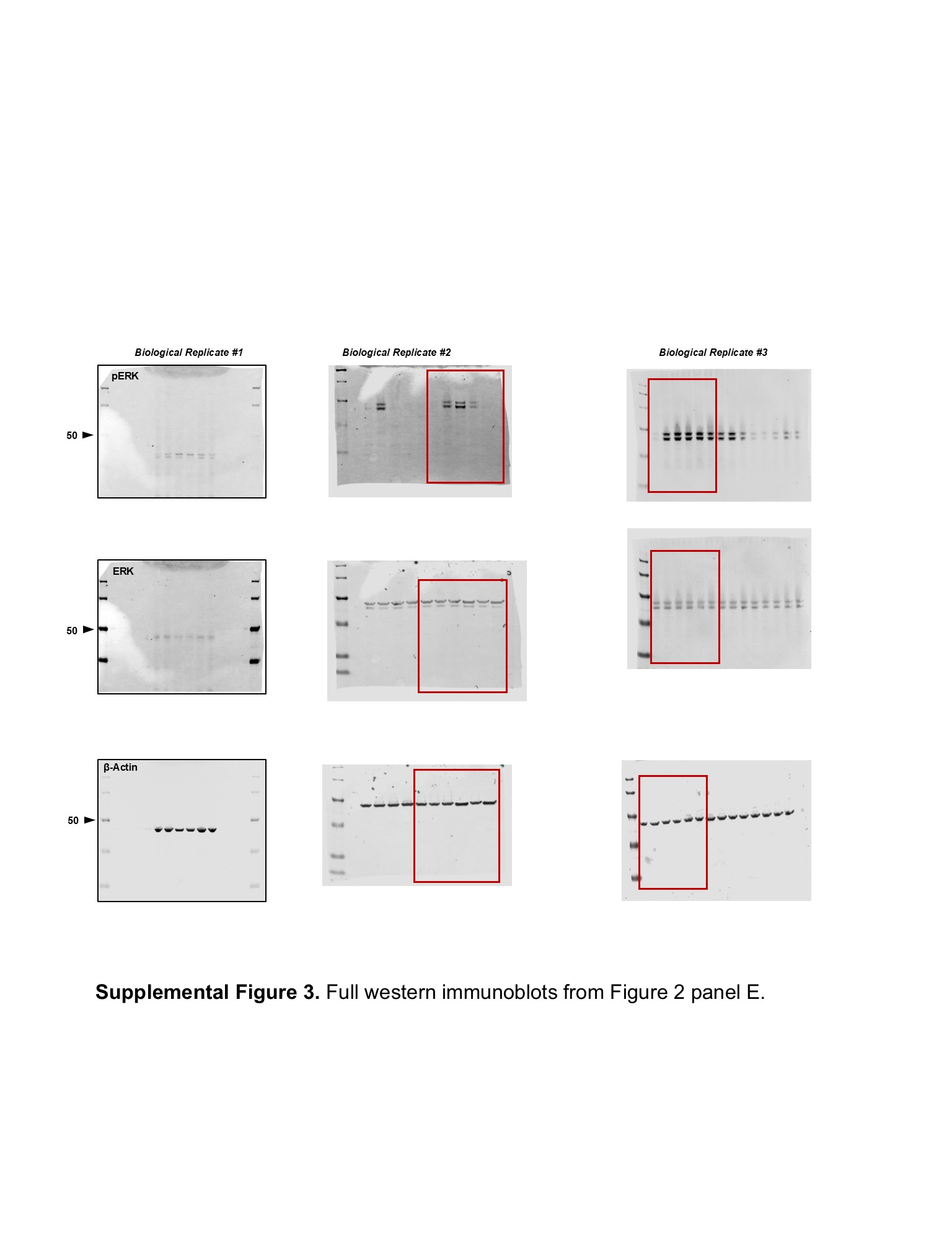

Supplement: Supplementary file 3 [file Image3.jpeg]

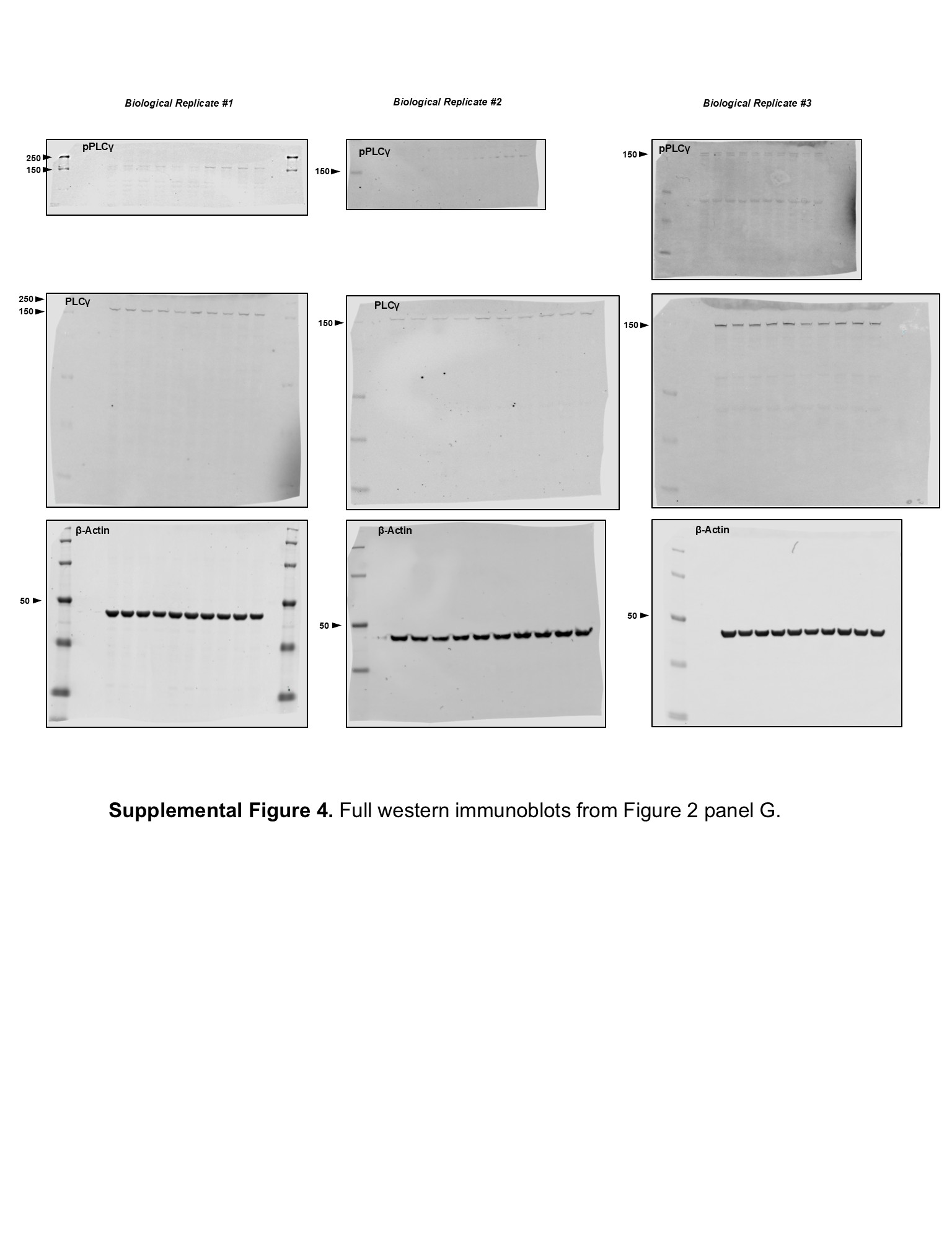

Supplement: Supplementary file 4 [file Image4.jpeg]

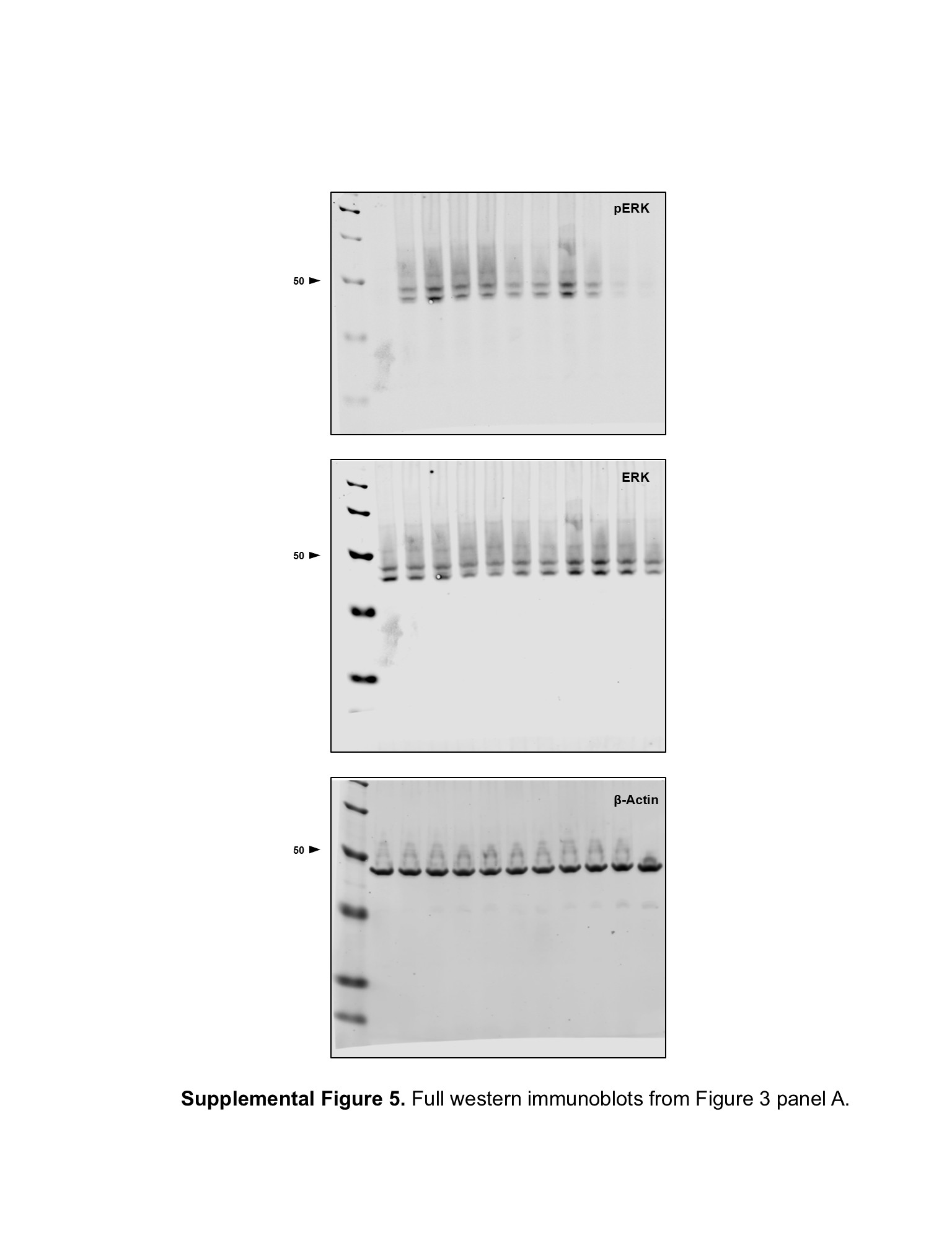

Supplement: Supplementary file 5 [file Image5.jpeg]

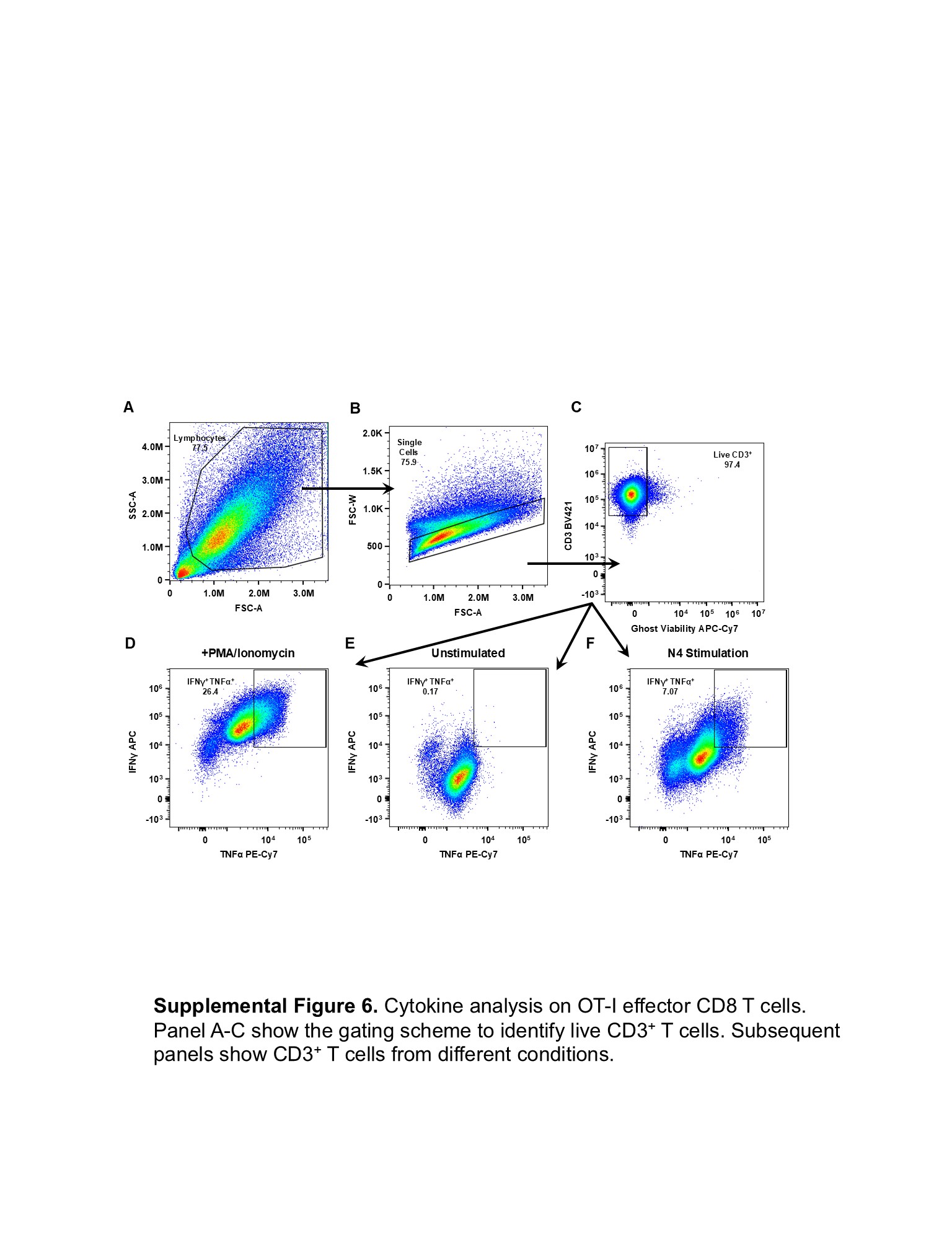

Supplement: Supplementary file 6 [file Image6.jpeg]

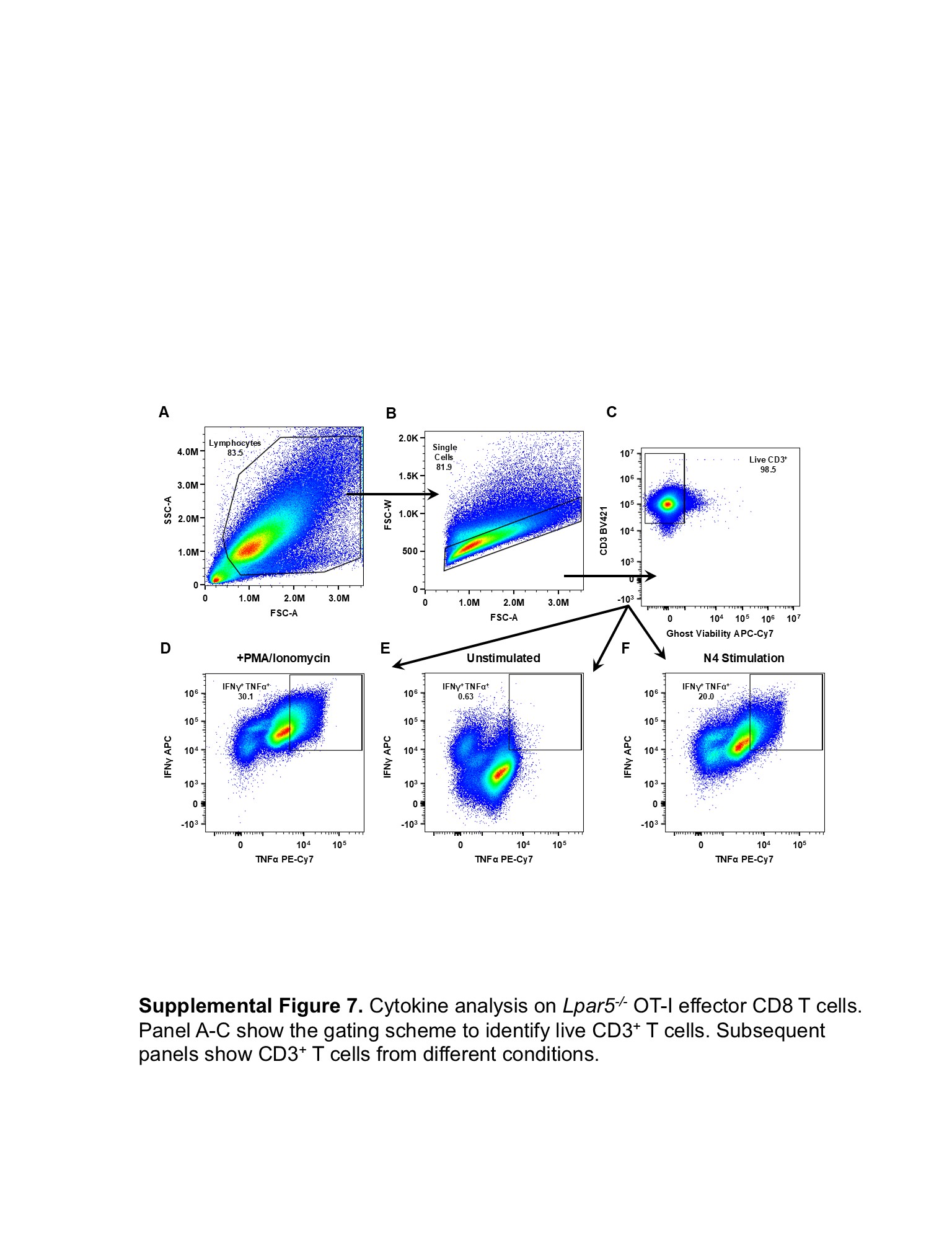

Supplement: Supplementary file 7 [file Image7.jpeg]
